# Targeting MGLL: terazosin regulates glycerolipid metabolism to mitigate endothelial cell senescence

**DOI:** 10.1016/j.jlr.2025.100904

**Published:** 2025-09-17

**Authors:** Jie Huang, Jinhua Yan, Zixin Wan, Tianyi Ji, Han Li, Wukaiyang Liang, Yi Huang, Zhen Yang, Yue Xiao, Hao Nie, Cuntai Zhang

**Affiliations:** 1 Department of Geriatrics, Tongji Hospital, Tongji Medical College, Huazhong University of Science and Technology, Wuhan, China; 2 Key Laboratory of Vascular Aging, Ministry of Education, Tongji Hospital, Tongji Medical College, Huazhong University of Science and Technology, Wuhan, China

**Keywords:** terazosin, endothelial cell senescence, monoglyceride lipase, palmitic acid, vascular aging

## Abstract

Metabolic disorders often arise in senescent endothelial cells, which impair endothelial function, lead to diminished vasodilation, increase vascular stiffness, and ultimately contribute to CVD pathogenesis. Despite notable advancements, the molecular mechanisms driving endothelial senescence and its contribution to vascular aging remain incompletely understood, thereby limiting the development of effective therapeutic strategies. Here, we investigated the protective role of terazosin (TZ) against vascular endothelial senescence using both in vivo (aged mice) and in vitro (human umbilical vein endothelial cells) models, combined with senescence-associated β-galactosidase staining, lipidomics, and molecular docking simulations. TZ treatment significantly improved endothelium-dependent vasodilation, reduced vascular stiffness, and attenuated the expression of senescence markers in aged mice. Mechanistically, lipidomics revealed that TZ reduced intracellular palmitic acid (PA) accumulation in senescent endothelial cells. Furthermore, clinical observations confirmed decreased plasma PA levels and improved endothelial function in patients receiving TZ. Monoglyceride lipase (MGLL), which hydrolyzes monoglycerides into PA and glycerol, was markedly upregulated in senescent endothelial cells and aged vascular tissues. TZ directly bound to MGLL and inhibited its enzymatic activity, thereby mitigating PA-driven endothelial senescence. Collectively, these findings identify MGLL as a novel metabolic driver of endothelial senescence and establish TZ as a potential therapeutic agent for age-related vascular diseases.

## Graphical abstract

**Figure:**
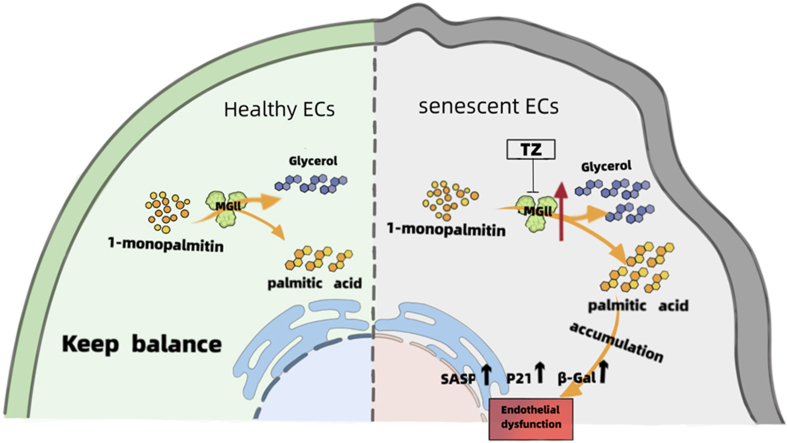

CVDs remain the leading global cause of mortality, with aging being the most significant risk factor (1). Vascular aging, a hallmark of systemic aging, is characterized by endothelial dysfunction and strongly contributes to the onset and progression of CVDs. Cellular senescence is a cellular program characterized by stable growth arrest and phenotypic changes, including chromatin remodeling, metabolic reprogramming, increased autophagy, and the secretion of proinflammatory mediators (senescence-associated secretory phenotype) (2, 3). Dysfunctional endothelium results in impaired endothelium-dependent dilation (EDD) and increased arterial stiffness, both of which are predictive of clinical CVDs and contribute to their pathophysiology (4, 5, 6). Endothelial dysfunction is one of the earliest detectable abnormalities associated with CVD, serving as a precursor and predictor of subsequent disease development and related mortality (4). Emerging evidence suggests that senescent endothelial cells drive endothelial dysfunction, thereby increasing the risk of age-related vascular diseases (7, 8, 9). Consequently, elucidating the molecular mechanisms governing endothelial senescence is critical for developing preventive strategies against vascular aging and associated disorders.

Endothelial cells, forming the inner lining of blood vessels, play a central role in vascular function by directly interacting with circulating blood components. Endothelial senescence can be triggered by diverse stressors (10), such as oxidative stress (11), replicative exhaustion (12), disturbed flow (13, 14), and inflammatory cytokines (15). Emerging evidence also suggests that the vascular endothelium is a critical regulator of systemic metabolic homeostasis (16, 17). Dysregulated levels of insulin, glucose, amino acids, and lipids, resulting from physiological dysfunction or pathological states, can lead to endothelial senescence, which in turn contributes to disease progression (13). Among these factors, hyperlipidemia and fatty accumulation have emerged as key drivers of vascular aging. Lipotoxicity, caused by excessive FFAs, is thought to induce endothelial senescence through increased oxidative stress (18). FFA-mediated endothelial dysfunction involves complex mechanisms, including impaired insulin signaling, reduced nitric oxide (NO) production, heightened oxidative stress, inflammation, activation of the renin-angiotensin system, and endothelial apoptosis (19). In particular, exposure to high concentrations of palmitic acid (PA), the most abundant saturated FFA in human plasma, promotes endothelial dysfunction and impairs endothelium repair by activating an inhibitor of nuclear factor-kappa-B kinase subunit beta (20), a crucial signaling pathway implicated in cellular senescence (21). Despite these advancements, the mechanisms underlying FFA dysregulation and FFA-induced lipotoxicity in aging endothelial cells remain inadequately understood. This knowledge gap underscores the necessity of developing novel therapeutic strategies targeting FFA-related signaling pathways to effectively alleviate endothelial dysfunction and its complications, such as atherosclerosis.

Terazosin (TZ), an α_1_-adrenergic receptor antagonist commonly prescribed for benign prostatic hyperplasia (BPH), exhibits therapeutic potential beyond its conventional applications. Recent studies have revealed that TZ enhances glycolysis and increases cellular ATP levels by binding to the enzyme phosphoglycerate kinase 1 (22). Furthermore, by elevating brain ATP levels in a heat shock protein 90-dependent manner, TZ has been reported to slow neurodegeneration not only in Parkinson's disease (23) but also in models of Alzheimer's disease (24). Additionally, the anti-inflammatory and antiapoptotic properties of TZ have shown protective effects against abdominal aortic aneurysm formation in mice (25). TZ also increases the activity of LDL receptors, promotes the transport and metabolism of LDL, and enhances lipid metabolism (26, 27, 28). Although TZ has been observed to lower plasma lipid levels (29), the mechanism underlying its effects on lipid metabolism in the aged vascular endothelium, if any, has not been elucidated to date.

Maintaining endothelial integrity is paramount for counteracting vascular aging. Our findings demonstrate that TZ treatment significantly improved endothelial function in patients with arteriosclerosis and in aged mice. Using surface plasmon resonance (SPR) and molecular docking approaches, we identified monoglyceride lipase (MGLL) as a novel target of TZ. Overexpression of *MGLL* in endothelial cells was notably associated with the development of vascular senescence, which was further validated in mouse models. Furthermore, TZ significantly reduced plasma PA levels in patients with arteriosclerosis, and subsequent experiments confirmed that TZ mitigates vascular aging by targeting MGLL to decrease PA accumulation in senescent endothelial cells.

## Materials and Methods

### Patients, specimens, and follow-up

Blood samples were obtained from the specimen archives of patients enrolled in a clinical trial conducted at Tongji Hospital, Tongji Medical College, Huazhong University of Science and Technology, between August 2023 and September 2024 (30). Flow-mediated dilation (FMD) data were collected from follow-up assessments. The study included participants aged over 40 years with elevated arterial stiffness, defined as a brachial-ankle pulse wave velocity ≥1,400 cm/s. Participants were administered TZ at doses of 0.5 or 1.0 mg/day during the study period. The selection of low-dose TZ (0.5 and 1.0 mg/day) was based on previous findings that both doses improve arterial stiffness without lowering blood pressure, demonstrating comparable efficacy. This supports the use of low, nonantihypertensive doses to investigate the vascular protective effects of TZ independent of its hypotensive action. Exclusion criteria included age <40 or >85 years; pre-existing CVDs; cancer; pregnancy; hepatic or renal dysfunction; acute infections; and patients requiring glucose- or lipid-lowering medications due to abnormal metabolic indices. We also excluded individuals receiving antihypertensive therapy and those unable to adhere to treatment or follow-up procedures to minimize confounding effects from non-TZ drugs affecting arterial stiffness. All study procedures were reviewed and approved by the Ethics Committee of Tongji Hospital, Huazhong University of Science and Technology (Wuhan, China), and adhered to the principles of the Declaration of Helsinki. The study protocol was registered at ClinicalTrials.gov (no. ChiCTR2000040605). Written informed consent was obtained from all participants.

### Animals

Young (2–3 months) and old (20–22 months) male C57BL/6J mice were purchased from Beijing HFK Technology (Beijing, China). The mice were certified to be free of specific pathogens. Mice were housed in a temperature-controlled barrier facility with a 12-h light/12-h dark cycle and had free access to food and water. All animal experiments were performed in accordance with the Regulations for the Administration of Affairs Concerning Experimental Animals, as drafted by the Chinese Ministry of Science and Technology. Animal experiments were approved (approval no.: TJH-202108010) by the Laboratory Animal Welfare and Ethics Committee of Tongji Hospital, Huazhong University of Science and Technology.

### Drug administration

**Fig. 1:**
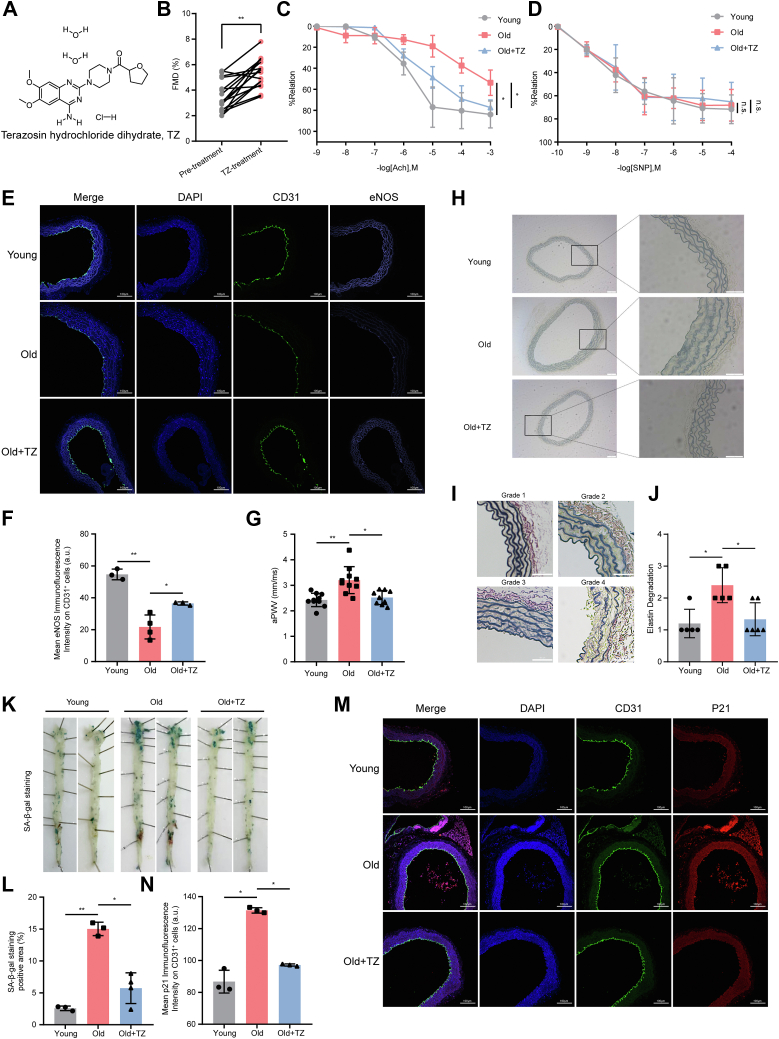
TZ improved vascular aging. A: Chemical structure of TZ hydrochloride dihydrate. B: Comparison of FMD in patients before and after TZ treatment (n = 16). C: ACh-induced endothelium-dependent vasodilatory function in the aorta rings of young, old, and old + TZ mice (n = 3). D: SNP-induced endothelium-independent vasodilatory function (n = 3). E: Representative immunofluorescent staining images of CD31 (green), eNOS (purple), and DAPI (blue) in aortas. F: Quantification of mean eNOS immunofluorescence intensity on CD31^+^ cells (a.u.) (n = 3–4). G: aPWV comparison (n = 9–10). H: Representative EVG staining images of the aortas. I: Elastin degradation grading criteria. J: Quantification of the elastin degradation score in aortas (n = 5–6). K: SA-β-gal staining of mouse aortas. L: Quantification of SA-β-gal staining positive area (n = 3–4). M: Representative immunofluorescent staining images of CD31 (green), p21 (red), and DAPI (blue) in aorta samples from young, old, and old + TZ groups. N: Quantification of mean p21 immunofluorescence intensity on CD31^+^ cells (a.u.) (n = 3). Error bars represent the mean ± standard deviation. Statistical analyses: paired *t*-test (B), unpaired *t*-test of Emax (C and D), and one-way ANOVA (F, G, J, L, and N). ∗*P* < 0.05, ∗∗*P* < 0.01. DAPI, 4',6-diamidino-2-phenylindole.

TZ (98% purity) was purchased from Sigma-Aldrich (T4680, St Louis, MO). Mice in the TZ-treatment group were administered TZ daily at a dosage of 100 μg/kg via oral gavage. The drug was dissolved in distilled water. In the control group, an equivalent volume of distilled water was administered in a similar manner. The dosage and administration route were selected based on our previous study, in which oral gavage of 100 μg/kg TZ effectively attenuated vascular remodeling in mice (25). The human equivalent dose was calculated as 0.0081 mg/kg (≈0.49 mg/day for a 60 kg human) using Food and Drug Administration body surface area normalization guidelines (human equivalent dose = animal dose × [Km mouse/Km human]; Km mouse = 3, Km human = 37), confirming alignment with the low-dose clinical regimen (0.5–1.0 mg/day). The chemical structure formula of TZ is illustrated in Figure 1A.

### Isolation and culture of human umbilical vein endothelial cells

Human umbilical vein endothelial cells (HUVECs) were isolated from human umbilical cords using collagenase digestion, as described previously (31). Umbilical cords were collected from donors (25–30 years old) with informed consent, as approved by the Human Experimentation Ethics Committee of Tongji Hospital, Huazhong University of Science and Technology (approval no.: TJ-IRB20230419). HUVECs were cultured in T25 flasks using ECM (ScienCell Research Laboratories, Carlsbad, CA) supplemented with 5% FBS, 100 mg/ml streptomycin/100 U/ml penicillin, and 1% endothelial cell growth supplement. Cells were cultured in a constant temperature incubator at 37°C with a 5% CO_2_ atmosphere and subcultured with 0.05% trypsin-EDTA (Gibco) at 80% confluence. A replicative senescence model was established by consecutively passaging young HUVECs until cell proliferation was nearly arrested. Young (passages 5–12, p5–p12) and senescent (passages 20–26, p20–p26) HUVECs were used for experiments. For senescence induction experiments, HUVECs were treated with 100 μM PA (Sigma-Aldrich), a concentration established in prior literature demonstrating its ability to induce endothelial senescence in vitro (32). MG 16:0/0:0/0:0 (1-monopalmitin; TargetMol) was used at 100 μM, based on IC_50_ determined in HUVECs using the Cell Counting Kit-8 assay. Both compounds were administered for 24 h prior to subsequent analyses.

### Quantitative lipid profiles

We meticulously harvested freshly cultured HUVECs from p5, p21, and p21 + TZ groups, subsequently transferring them to cold 1× PBS. Cell pellets containing at least 1 × 10^6^ cells per sample in vitro were subjected to liquid extraction. Ultra-performance liquid chromatography and MS/MS analysis were performed at Wuhan Metware Biotechnology Co, Ltd (Wuhan, China). Protein concentration was measured using a BCA Protein Assay kit, and the metabolomic data of the detection samples were calibrated with the protein concentration of parallel samples. The complete dataset, including all detected lipid species and their normalized abundances, is provided in supplemental Table S1.

### Quantification of intracellular PA

Cellular PA levels were quantified by GC-MS. Briefly, cells were collected, and total lipids were extracted using a methyl tert-butyl ether-based method. Lipids were then methylated to generate fatty acid methyl esters, which were analyzed using an Agilent 8890B GC system coupled to a 7000E mass spectrometer (Agilent Technologies). Fatty acid methyl esters were separated on a DB-5MS capillary column (30 m × 0.25 mm × 0.25 μm), and PA was identified and quantified using authentic standards. All values were normalized to total protein content. The complete dataset, including all detected FFAs and their normalized abundances, is provided in Supplemental FFAs Raw Intensities.

### Senescence-associated-β-galactosidase staining

Senescence-associated-β-galactosidase (SA-β-gal) staining was performed to observe morphological changes of senescent vascular tissues and senescent cells using the senescence β-galactosidase staining kit (Abbkine, Wuhan, China) according to the manufacturer's instructions.

Vascular tissues were fixed with 4% buffered paraformaldehyde for 30 min at room temperature, rinsed with PBS twice, and incubated for 16 h at 37°C with β-gal staining solution. After washing, images were analyzed using ImageJ software to quantify the SA-β-gal-positive proportion area.

HUVECs were fixed with 4% buffered paraformaldehyde for 15 min at room temperature, rinsed with PBS twice, and incubated for 6 h at 37°C with β-gal staining solution. Cells were washed with cold PBS to cease staining. ImageJ was used to count blue-stained cells and total cells. The percentage of β-galactosidase-positive cells was calculated.

### FMD measurement

Endothelial function was assessed by brachial artery FMD using the UNEX EF18VG system (UNEX Co, Nagoya, Japan) equipped with an H-type linear array transducer, as previously described (33, 34). All participants fasted for at least 8 h and refrained from caffeine, nicotine, and alcohol for at least 12 h prior to the assessment. After 15 min of supine rest in a temperature-controlled room (23 ± 1°C), brachial artery diameter changes were recorded during the measurement protocol. Two primary diameter measurements were obtained: the baseline diameter (D_0_) and the peak dilation diameter (D_max_). FMD was calculated using the following formula: FMD (%) = [(D_max_ - D_0_)/D_0_] × 100.

### Assessment of aortic pulse wave velocity

Aortic pulse wave velocity (aPWV) was assessed using a Doppler Flow Velocity System (Indus Instruments, Houston, TX), following the manufacturer's instructions. Briefly, each mouse was placed in a supine position and connected to electrocardiogram electrodes on a heated board (37°C). A Doppler probe recorded the flow velocity signals from both the aortic arch and abdominal aorta. The distance between the probe tips was measured using calipers. PWV was calculated using the transit time method as follows: PWV (m/s) = (distance between probe tips)/(time between the start of the wave at position 1 and the start of the wave at position 2). The average aPWV was determined from three separate measurements to obtain the final aPWV value.

### Assessment of EDD

The isolated aortic functional changes were assessed by evaluating the EDD ability of thoracic aorta rings using an in vitro microvascular tone measurement system (DMT, Denmark). The measurement of vascular reactivity in the mouse thoracic aorta has been previously described (35). In brief, an intact thoracic aortic ring (2 mm) was mounted on an isometric wire myograph (DMT620M; ADInstruments, Australia) filled with physiological saline solution aerated with 95% O_2_ and 5% CO_2_. The aortic ring was gradually stretched to an optimal baseline tension of 5 mN while equilibrating for 60 min. The aortic ring was constricted three times with 60 mM KCl, and the maximum amplitude of the contractile response was similar. Subsequently, the thoracic aorta was constricted by the addition of 3 × 10^-6^ M phenylephrine (Sigma-Aldrich). To assess endothelium-dependent vasodilatation, relaxation was achieved by cumulative addition of acetylcholine (ACh, 10^-9^–10^-3^ M; Sigma-Aldrich) at a maximum amplitude. The arteries were then rewashed three times; after 30 min incubation, the arteries were contracted by 3 × 10^-6^ M phenylephrine, and the endothelium-independent vasorelaxation induced by different concentrations (10^-9^–10^-4^ M) of sodium nitroprusside (Sigma-Aldrich) was measured. The cumulative dose-response curve was collected using the supporting software of the DMT system. For statistical analysis, maximal relaxation (E_max_) at the highest ACh concentration was compared between groups using an unpaired two-tailed Student’s *t*-test, as this parameter reflects the peak endothelium-dependent vasodilatory capacity.

### Elastica van Gieson staining

Mice were perfused with 0.9% saline and 4% paraformaldehyde for immunohistochemical observation. Thoracic-abdominal aorta was paraffin-embedded and sliced into 4-μm-thick tissue sections. The artery tissue sections were deparaffinized with xylene and hydrated in ascending grades of ethanol. Next, the slides were incubated with Elastica van Gieson solution for half an hour (a mixture of hematoxylin, iodine solution, and ferric chloride at a ratio of 5:2:2). The background was differentiated with ferric chloride. The artery tissue sections were redyed with VG solution (a mixture of saturated picric acid and fuchsin solution at a ratio of 9:1) and dehydrated with ethanol. All stained artery slides were photographed under a light microscope (Nikon, Japan). The degradation scoring standard was employed as previously described (36). A grade 1 score signifies a normal medial layer with no signs of elastic strand breaks or <25% degradation; a grade 2 score represents mild disruption in the medial layer of the elastic network, with 25–50% degradation; a grade 3 score indicates severe elastin degradation, ranging from 50% to 75%; and a grade 4 score was assigned when all the elastic layers exhibited signs of breakage or degradation were >75%.

### Immunofluorescence staining

The paraffin-embedded sections of the thoracic aorta were stained by immunofluorescence. In brief, the sections were dewaxed with xylene and gradient alcohol before being repaired for 2 min at high temperature and pressure with EDTA antigen retrieval solution (pH 9.0). The sections were washed thrice with Tris-buffered saline containing Tween-20 (TBST) buffer and treated with 3% H_2_O_2_ at room temperature for 30 min. After being immersed in distilled water, the sections were incubated with 10% goat serum at 37°C for 30 min to quench endogenous peroxidase activity. The serum was discarded, and the primary antibody anti-CD31 (ab182981, 1:200 dilution; Abcam) was added and left at 4°C overnight. After washing with TBST, the secondary antibody Goat Anti-Rabbit IgG H&L (HRP; ab2057188, 1:4,000 dilution; Abcam) was incubated at 37°C for 45 min. The working solution of iFluor 488 tyramide (#11060, 1:300 dilution; aatbio) or Cy5-tyramide (#11066, 1:400 dilution; aatbio) was then incubated at room temperature for 10 min. After elution and blocking again, primary antibodies against MGLL (ab307162, 1:300 dilution; Abcam), p21 (sc-6246, 1:100 dilution; Santa Cruz Biotechnology, TX) and secondary antibodies were incubated and stained with iFluor 488 tyramide or Cy5-tyramide. The nucleus was stained, and the sections were sealed with an antifade mounting medium (containing 4′,6-diamidino-2-phenylindole). The sections were visualized and imaged using fluorescence confocal microscopy (Nikon C2).

### Screening of TZ-related targets

To identify potential targets for TZ, we utilized the Swiss TargetPrediction web server (http://www.swisstargetprediction.ch/) (37). Subsequently, we standardized the target protein symbols to align with the UniProt protein database (https://www.uniprot.org/) (38).

### Molecular docking analysis

The crystal structure of MGLL (Protein Data Bank ID: 5ZUN) was obtained from a protein database and subjected to energy minimization to prepare the receptor structure for molecular docking. The structure of TZ was constructed, hydrogenated, and structurally optimized using a molecular orbital package program. Molecular docking was performed using AutoDock Vina software (version 1.2.x) from the Molecular Graphics Laboratory at Scripps Research (La Jolla, CA), with the docking box encompassing the active site. All other parameters were set to their default values.

### Molecular dynamics simulation and analysis

Molecular dynamics (MD) simulations of MGLL and TZ were performed using the Desmond program. The OPLS2005 force field was employed to parameterize the interactions between MGLL and TZ. The TIP3P water model was used to solvate the MGLL-TZ system within a water box, and a sodium ion equilibrium system was added. Before initiating the simulation, the system energy was minimized in 50,000 steps using the steepest descent method to ensure a relatively stable initial state. Subsequently, canonical (NVT) and isothermal-isobaric (NPT) equilibrium systems were employed, followed by a 100 ns MD simulation conducted at room temperature and pressure to observe the dynamic behavior of MGLL-TZ complexes in the free state. Throughout the simulation, the system temperature was maintained at 300 K, and the pressure was kept at 1 bar to mimic physiological conditions. Based on the MD simulation’s trajectory data, the stability of each system was assessed by using root mean square deviation, root mean square fluctuation (RMSF), and ligand-protein contact analysis (39).

### Virtual amino acid mutation analysis

According to the binding mode of MGLL and TZ, all amino acids within 5 Å of the small molecules in the MGLL-TZ complex were selected as scanning objects. Using Schrödinger's Residue Scanning module, these amino acids were scanned by alanine mutagenesis; that is, they were mutated to alanine one by one. The changes in binding energy before and after mutation were calculated to evaluate the importance of each amino acid residue for the binding of TZ and MGLL.

### SPR analysis

SPR analysis was run on a Biacore T200 system (Cytiva) with a Series S Sensor Chip CM5. Experiments were performed by TGTMED MediTech Co, Ltd (Shanghai, China). Human recombinant MGLL protein was purchased from Cayman Chemical (10007812; Cayman Chemical, Ann Arbor, MI). Briefly, the ligand (MGLL) was immobilized to the CM5 Chip via covalent bonds to the amino acid residues in the immobilization buffer. Subsequently, different concentrations of TZ were diluted in the analyte buffer and were injected into the flowing channel to allow interaction between MGLL and TZ. The interacting phase included 120 s of association phase and 240 s of dissociation phase. The data were analyzed using Biacore T200 Evaluation Software (Cytiva).

### Human Protein Atlas and UniProt database analysis

The Human Protein Atlas (HPA) database (http://www.proteinatlas.org/) was used to obtain the mRNA level of *MGLL* in different cell lines, a summary of normalized single-cell RNA (nTPM) levels of *MGLL* across all single cell types, and single-cell RNA levels of *MGLL* specifically within vascular tissue (40). The cellular location of MGLL was identified using the UniProt database (https://www.uniprot.org/) with a subcellular map (38).

### Transient siRNA transfection

The cells were transfected with siRNA using Lipofectamine 3,000 (Thermo Fisher Scientific) according to the manufacturer’s instructions for 48 h. siRNA specific for *MGLL* and control siRNA were obtained from General Biol (Anhui, China). The targeting sense sequences are 5′-GGUCGACAUUUAUAACUCATT-3′ (si*MGLL*#1) and 5′-UCGCCUAUGUGACAGCAAATT-3′ (si*MGLL*#3). The control siRNA sequence is 5′-UUCUCCGAACGUGUCACGUTT-3′.

### Lentiviral overexpression of *MGLL*

Lentiviral production was purchased from GeneChem (Shanghai, China). The lentivirus for *MGLL* overexpression was named "MGLL-OE," whereas the control lentivirus was named "Vec." HUVECs (p5) were transfected with the lentivirus using the manufacturer's protocol. The vector name was "GV492," and the element order was "Ubi-MCS-3FLAG-CBh-gcGFP-IRES-puromycin." A mixture of virus diluent (multiplicity of infection = 100), HitransG, and complete medium was allowed to stand for 10 min before being added to HUVECs. Notably, the complete medium was replaced 8 h post-transfection, and puromycin at 0.5 μg/ml was used for screening 72 h post-transfection. The efficiency of gene overexpression was confirmed using RT-qPCR and Western blotting (WB).

### RNA extraction and RT-qPCR analysis

Total RNA was purified by RNA purification kit (Magen, China) and reverse-transcribed using the HiScript RT Kit (R222; Vazyme, Nanjing, China) following the manufacturer's instructions. RT-qPCR was performed using a Light Cycler 480 II instrument (Roche, Mannheim, Germany) and a ChamQ SYBR qPCR Mix (Q711; Vazyme). Each assay was performed at least three times. Gene expression levels were calculated using the 2−ΔΔCt method, with 18S rRNA serving as an endogenous control.

### Western blot

Cells were lysed in RIPA buffer (Beyotime Institute of Biotechnology, Haimen, China) supplemented with a proteinase and phosphatase inhibitor cocktail (Hoffmann-La Roche Ltd, Basel, Switzerland) for 1 h on ice. The protein concentration was determined using Pierce™ BCA Protein Assay Kits (Thermo Fisher Scientific, Inc, Carlsbad, CA). A total of 20 μg of each protein was separated by 10% SDS-PAGE (Boster Biotechnology, Wuhan, China) and transferred to a polyvinylidene difluoride membrane (Millipore, Billerica, MA). Membranes were blocked with 5% skim milk dissolved in 1X TBST. Then, the membranes were incubated with primary antibodies against PAI-1 (66261-1-Ig, 1:1,000 dilution; Proteintech, Wuhan, China), β-tubulin (66240-1-Ig, 1:20,000 dilution; Proteintech), and p21 (sc-6246, 1:500 dilution; Santa Cruz Biotechnology, TX) at 4°C overnight. Subsequently, the membranes were incubated with HRP-conjugated anti-rabbit or anti-mouse secondary antibodies (1:5,000 dilution; Jackson ImmunoResearch Laboratories, Inc, West Grove, PA) for 1 h at room temperature. Detection was performed using a ChemiDoc™ Imaging System (Bio-Rad Laboratories, Inc, Hercules, CA).

### Statistical analysis

Data were expressed as means ± SEM from at least three independent experiments. Statistical analysis was performed using Prism 8.0 (GraphPad Software, Inc, La Jolla, CA). For comparisons between two independent groups, unpaired two-tailed Student’s *t*-tests were used (or Mann-Whitney *U* tests when non-normal). For paired measurements, paired two-tailed *t*-tests were applied. For multiple-group comparisons, one-way ANOVA followed by Tukey's post hoc test was used. Statistical significance was defined as *P* < 0.05.

## Results

### TZ treatment ameliorates vascular aging in vivo

The chemical structure of TZ, an α_1_-adrenergic receptor antagonist primarily prescribed for BPH and hypertension (41), is shown in Figure 1A. Beyond its established clinical use, TZ has also been reported to exert broader therapeutic effects, including in Parkinson's disease (23) and abdominal aortic aneurysm (25). To explore its potential vascular impact, we first conducted a pilot clinical study in patients aged >40 years with elevated arterial stiffness (brachial-ankle pulse wave velocity ≥1,400 cm/s). Participants received low-dose TZ (0.5 or 1.0 mg/day) for 6–12 months between August 2023 and September 2024. Both doses were selected to minimize blood pressure reduction while maintaining efficacy (30). Notably, no differential outcomes were observed between doses, confirming that a pooled analysis was appropriate. Sixteen participants underwent FMD, a noninvasive gold-standard method to evaluate endothelial function based on measuring arterial dilation responses (42, 43). This assessment revealed a significant improvement in endothelial function after TZ administration (Fig. 1B). Endothelial dysfunction is a key manifestation of vascular aging (44). To further investigate whether TZ could improve age-related endothelial dysfunction and vascular remodeling, 19- to 20-month-old male mice were treated with TZ (100 μg/kg/day, via oral gavage) or vehicle for 3 months. Vascular endothelial dysfunction and large elastic artery stiffness were evaluated as key predictors of vascular aging (45). Ex vivo vascular ring tension assays showed that aged mice exhibited impaired ACh-induced endothelium-dependent vasodilatory function compared with young controls, which was significantly restored by TZ, whereas sodium nitroprusside-induced endothelium-independent vasodilatory function remained unchanged (Fig. 1C, D). Consistently, TZ treatment reversed the age-related decline in endothelial NO synthase expression, the key enzyme responsible for producing vasodilatory NO (46) (Fig. 1E, F). TZ also attenuated the aging-associated increase in aPWV (Fig. 1G), a measure of large elastic artery stiffness (45), and preserved the integrity of elastic laminae in aortic segments, as evidenced by Elastica van Gieson staining (Fig. 1H, J). These findings suggest that TZ exerts protective effects against aging-related endothelial dysfunction and vascular remodeling.

To further assess TZ’s impact on vascular senescence, SA-β-gal staining of the aorta revealed a pronounced increase in senescence-associated positive areas in aged mice, which was significantly mitigated by TZ treatment (Fig. 1K, L). Immunofluorescence staining of paraffin-embedded aortic tissue sections further confirmed that TZ treatment decreased the expression of endothelial aging marker p21, which was elevated with age (Fig. 1M, N). Collectively, these results demonstrate that TZ treatment mitigates vascular aging by alleviating vascular remodeling and endothelial dysfunction associated with aging.

### TZ regulates glycerolipid metabolism in senescent vascular endothelial cells

**Fig. 2:**
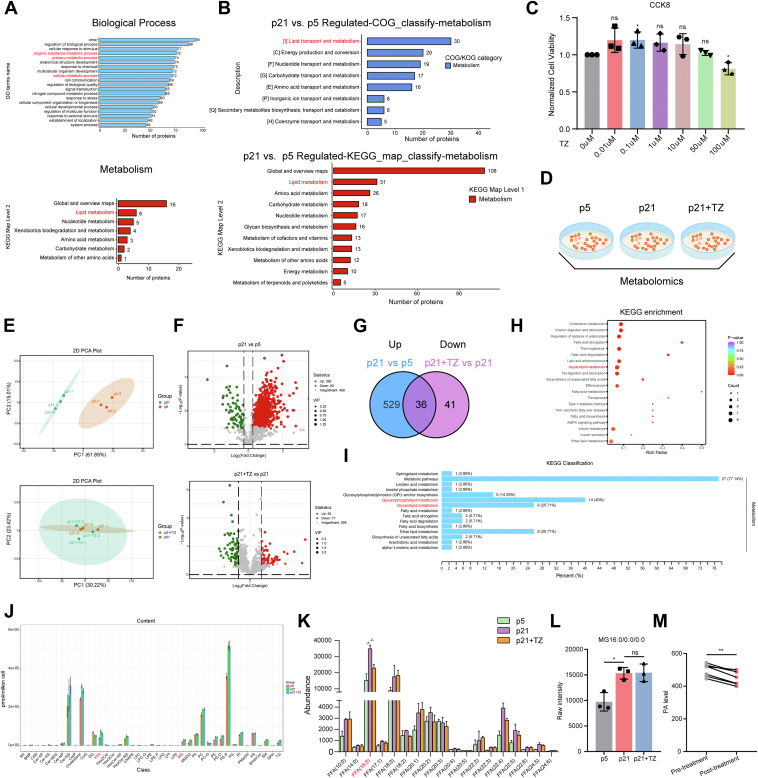
Analysis of the effects of TZ on lipid metabolism in senescent endothelial cells. A: Functional classification of TZ targets in Gene Ontology Biological Processes (GO-BP) and KEGG metabolic pathways. B: Functional classification of differentially expressed genes (fold change >1.3, CV < 0.1; n = 2) in p21 versus p5 from proteomic analysis within COG and KEGG metabolic pathways. C: Cell Counting Kit-8 assay to assess the effect of TZ on the viability of HUVECs. D: Schematic diagram of the lipid metabolomics study design. E: Principal component analysis (PCA) plot illustrating sample clustering. F: Volcano plots showing differential lipid metabolites between p21 versus p5 and p21 + TZ versus p21. G: Venn diagram identifying 36 metabolites upregulated in senescence but reduced by TZ. H: KEGG enrichment analysis of the 36 metabolites that are upregulated with aging and downregulated by TZ treatment. I: KEGG classification analysis of the 36 aging-upregulated and TZ-downregulated metabolites. J: Comparison of lipid subclass content across the three groups. K: Analysis of FFA subclass content across the three groups (n = 3). L: Analysis of MG 16:0/0:0/0:0 levels among the groups. M: Plasma PA levels in blood samples from patients receiving TZ treatment (n = 7). Error bars represent the mean ± standard deviation. Statistical analyses: unpaired *t*-test (C), one-way ANOVA (K, L), and paired *t*-test (M). ∗*P* < 0.05, ∗∗*P* < 0.01.

Recent studies suggest that TZ may influence cellular processes, such as metabolism (22) and senescence (25), although the exact mechanisms remain unclear. To identify potential targets of TZ, we searched the Swiss TargetPrediction database using "Terazosin" as the keyword, yielding 102 potential human targets, including previously validated ones. Gene Ontology analysis categorized these targets primarily into the regulation of biological processes, cellular response to stimulus, and metabolic processes. Kyoto Encyclopedia of Genes and Genomes (KEGG) pathway analysis further highlighted lipid metabolism as the predominant pathway among metabolic categories (Fig. 2A). Because endothelial cell senescence is a key driver of vascular aging (47) and is closely linked to metabolic disturbances (48), we next explored lipid metabolism in a replicative senescence model. Proteomic analysis revealed that differentially expressed proteins (fold change >1.3, CV < 0.1) were enriched in lipid metabolism pathways, both in metabolic categories within the Clusters of Orthologous Groups and KEGG pathway classification (Fig. 2B). These results point to lipid metabolism as a central process in endothelial senescence and a potential target of TZ. A preliminary Cell Counting Kit-8 assay confirmed that TZ was nontoxic to HUVECs at concentrations below 50 μM (Fig. 2C), and 100 nM was chosen for subsequent experiments.

To directly examine TZ’s effects on endothelial lipid metabolism, we performed lipid metabolomics on three groups: young HUVECs (p5), replicative senescent HUVECs (p21), and TZ-treated replicative senescent HUVECs (p21 + TZ) (Fig. 2D). Principal component analysis macroscopically reflected metabolic separation among the three groups (Fig. 2E). Employing a fold-change threshold of ≥1.2 and a variable importance in projection score >1 as criteria, the volcano plot illustrated that 89.6% of these differentially expressed lipid molecules were upregulated in senescent HUVECs, whereas 55.0% of these differentially expressed lipids were downregulated by TZ (Fig. 2F). Importantly, 36 differential metabolites that were upregulated during senescence were significantly reduced by TZ treatment (Fig. 2G). KEGG enrichment and classification analysis of these 36 metabolites highlighted glycerolipid metabolism (Fig. 2H, I). Broad lipid remodeling was observed during senescence: seven acylcarnitines and 57 triacylglycerols (TGs) were upregulated, whereas multiple diacylglycerols (DGs) were downregulated. TZ treatment selectively reduced seven TG species, including TG(16:0_18:0_20:4) and TG(16:0_18:1_20:4) but did not significantly alter acylcarnitine or DG levels (supplemental Table S2). Notably, monoacylglycerols (MAGs) were consistently upregulated in senescent cells, whereas no MAGs were significantly changed after TZ treatment. In contrast, FFAs were also significantly increased during endothelial senescence and were partially reduced by TZ (Fig. 2J). As emphasized by Madiraju *et al.* (49), the glycerolipid/FFA cycle plays a crucial role in numerous cellular signaling processes, including aging. Since MAGs are upstream substrates of FFAs (49), the concomitant increase in saturated MAGs and FFAs suggests that enhanced MAG hydrolysis may contribute to FFA accumulation in senescent cells. Among FFAs, PA was the most abundant and increased markedly with aging, whereas TZ effectively reduced its accumulation (Fig. 2K). Consistently, the level of its upstream lipid MG 16:0/0:0/0:0 was elevated during senescence but remained unchanged after TZ intervention (Fig. 2L). In line with the accumulation of PA, 19 palmitate-containing phosphatidylcholines and 10 palmitate-containing phosphatidylethanolamines were significantly increased in senescent cells, but none of these species were altered by TZ treatment (supplemental Table S3). This finding suggests that TZ preferentially regulates free PA rather than complex phospholipid-bound palmitate. Collectively, these findings indicate that endothelial senescence is accompanied by extensive lipid remodeling, with a notable accumulation of MAGs and FFAs. TZ selectively attenuates some of these lipid alterations, particularly by reducing PA, suggesting that regulation of glycerolipid metabolism may underlie its protective effects. Finally, to evaluate clinical relevance, we measured plasma PA levels in patients receiving TZ therapy. TZ significantly reduced circulating PA levels compared with baseline (Fig. 2M). Therefore, subsequent studies focused on the glycerolipid metabolic pathway as a potential mechanism through which TZ alleviates vascular endothelial aging.

### Relationship between glycerolipid metabolism and MGLL in senescent vascular endothelial cells

**Fig. 3:**
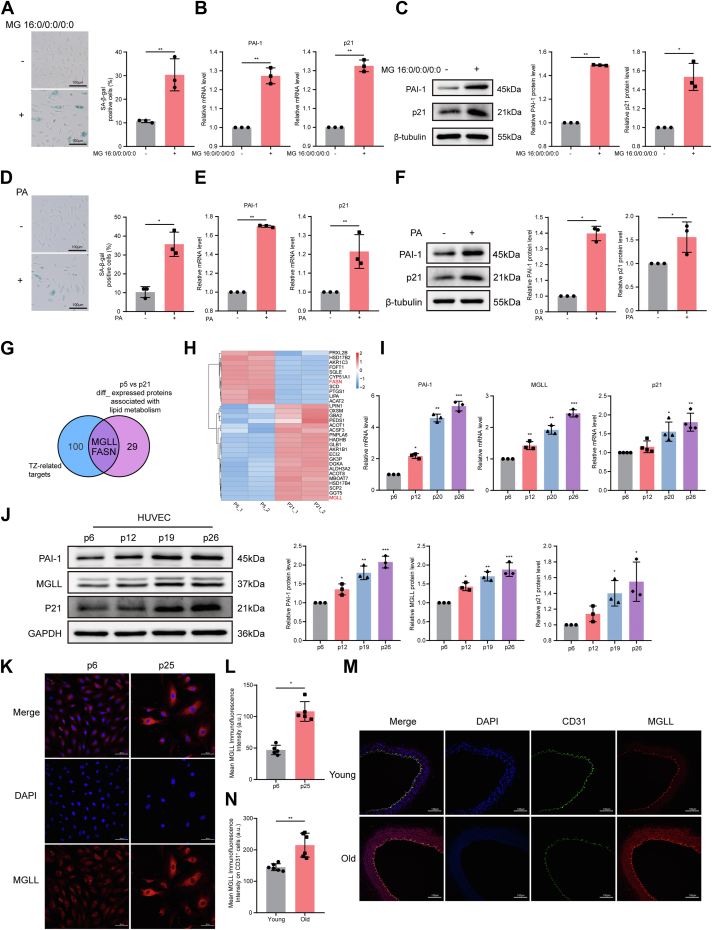
Glycerolipid metabolism and MGLL are closely associated with vascular endothelial senescence. A–C: Representative images and quantification of SA-β-gal staining (A), relative mRNA levels of *p21* and *PAI-1* by RT-qPCR (B), and representative Western blots with quantification of p21 and PAI-1 protein expression levels (C) in MG 16:0/0:0/0:0-treated HUVECs (n = 3). D–F: Representative images and quantification of SA-β-gal staining (D), relative mRNA levels of *p21* and *PAI-1* by RT-qPCR (E), and representative Western blots with quantification of p21 and PAI-1 protein expression levels (F) in PA-treated HUVECs (n = 3). G: Venn diagram illustrating the overlap between TZ target genes and differentially expressed lipid metabolism-related genes in HUVECs at passages 5 and 21 (FC > 1.3, CV < 0.1, n = 2). H: Heatmap of differentially expressed lipid metabolism-related proteins in HUVECs at passages 5 and 21 (FC > 1.3, CV < 0.1, n = 2). I and J: Relative mRNA (I) and protein (J) expression levels of MGLL, p21, and PAI-1 in HUVECs at passages 6, 12, 19, and 26 (n = 3). K: Representative immunofluorescence images of MGLL (red) and DAPI (blue) in HUVECs at passages 6 and 25. L: Quantification of mean MGLL immunofluorescence intensity in HUVECs at passages 6 and 25 (n = 3). M: Representative immunofluorescence images of MGLL (red), CD31 (green), and DAPI (blue) in aortas from young and old mice. N: Quantification of mean MGLL immunofluorescence intensity on CD31^+^ cells (a.u.) (n = 6). Error bars represent mean ± standard deviation. Statistical analyses: unpaired *t*-tests (A–F, I–J, L, and N). ∗*P* < 0.05, ∗∗*P* < 0.01, ∗∗∗*P* < 0.001. DAPI, 4′,6-diamidino-2-phenylindole.

Through in vitro experiments, including SA-β-gal staining, RT-qPCR, and WB, we confirmed that both MG 16:0/0:0/0:0 (a monoglyceride derived from glycerolipid metabolism) and PA induced endothelial cell senescence (Fig. 3A–F), suggesting a potential link between lipid accumulation and vascular aging. To elucidate the underlying mechanism, we focused on identifying critical enzymes that are regulated by TZ and that modulate glycerolipid metabolism during cellular aging. By intersecting TZ-associated targets with differentially expressed proteins associated with lipid metabolism (p5 vs. p21), we identified MGLL and FASN as critical candidate genes (Fig. 3G). In this respect, our proteomic analysis of differentially expressed proteins associated with lipid metabolism in a replicative senescence model revealed that MGLL, an enzyme that hydrolyzes MAGs into FFAs and glycerol (50), was significantly upregulated in senescent endothelial cells, whereas FASN, a key enzyme in de novo fatty acid biosynthesis, was downregulated (Fig. 3H). This observation suggests that senescent endothelial cells accumulate PA primarily through enhanced MAG hydrolysis rather than de novo synthesis.

Further validation in both cellular and animal models confirmed that MGLL expression increases with age in endothelial cells (Fig. 3I–N). Collectively, these findings highlight MGLL as a pivotal contributor to PA accumulation in senescent endothelial cells and implicate it as a potential driver of endothelial aging.

### Identification of MGLL as an endothelial cell senescence marker

**Fig. 4:**
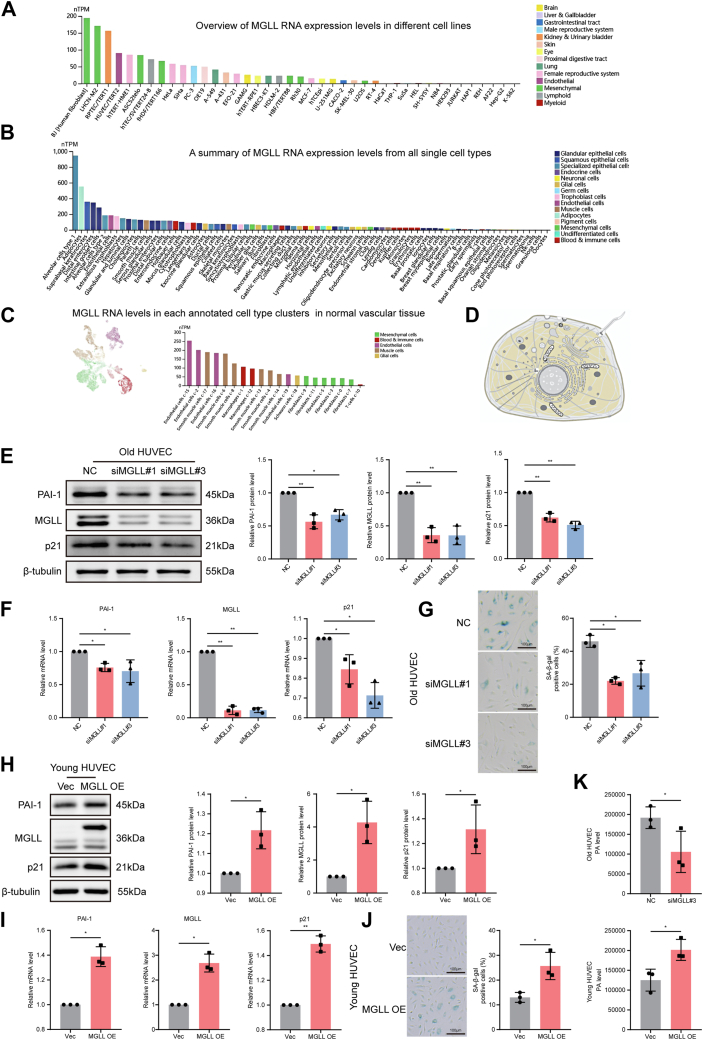
Expression and localization of MGLL and its role in endothelial cell senescence and PA metabolism. A: Overview of *MGLL* RNA expression levels in different cell lines (HPA). B: A summary of *MGLL* RNA expression levels from all single cell types (HPA). C: *MGLL* RNA expression levels in each annotated cell type of normal vascular tissue (HPA). D: Subcellular localization of MGLL in cell lines (UniProt). E and F: Representative Western blots (E) and RT-qPCR analysis (F) showing MGLL, p21, and PAI-1 expression levels in old HUVECs treated with *MGLL* siRNA (n = 3). G: Representative images and quantification of SA-β-gal staining in old HUVECs after *MGLL* knockdown (n = 3). H and I: Representative Western blots (H) and RT-qPCR analysis (I) showing MGLL, p21, and PAI-1 expression levels in young HUVECs transduced with *MGLL* lentivirus (n = 3). J: Representative images and quantification of SA-β-gal staining in *MGLL*-overexpressing young HUVECs (n = 3). K: Quantification of intracellular PA levels in HUVECs with *MGLL* overexpression or knockdown, measured by GC-MS (n = 3). Error bars represent the mean ± standard deviation. Statistical analyses: unpaired *t*-test (E–J) and paired *t*-test (K). ∗*P* < 0.05, ∗∗*P* < 0.01.

To assess the potential of MGLL as a marker of endothelial cell senescence, we systematically analyzed its mRNA expression and subcellular localization using the HPA and UniProt databases. *MGLL* RNA levels in HUVECs ranked among the top five across various cell lines (Fig. 4A). A broader single-cell transcriptomic analysis revealed that endothelial cells were among the top 10 cell types with the highest *MGLL* expression (Fig. 4B), with particularly high levels in endothelial cells within vascular tissue (Fig. 4C). Subcellular localization studies indicated that MGLL was predominantly localized to the plasma membrane and cytosol, supporting its functional relevance in these compartments (Fig. 4D). These bioinformatics analyses collectively suggest that MGLL plays a distinct role in endothelial cells.

To validate the functional role of MGLL experimentally, we performed siRNA-mediated knockdown and lentiviral overexpression in HUVECs. In HUVECs at p20, *MGLL* knockdown mediated by siRNA significantly reduced its expression compared with the negative control group (Fig. 4E, F). This was accompanied by decreased protein and mRNA levels of the senescence marker p21 and senescence-associated secretory phenotype factor PAI-1 (Fig. 4E, F), as well as reduced SA-β-gal activity (Fig. 4G). To further investigate the role of MGLL in senescence, we assessed the effects of lentivirus-mediated *MGLL* overexpression in young HUVECs (p5). The efficiency of overexpression was confirmed by the increased MGLL protein levels compared with the vector control group (Fig. 4H, I). In *MGLL*-overexpressing cells, both protein and mRNA levels of p21 and PAI-1 (Fig. 4H, I), as well as SA-β-gal activity (Fig. 4J), were significantly increased compared with the vector control group. Given MGLL’s role in glycerolipid metabolism, we further explored its impact on intracellular PA levels. GC-MS analysis revealed that *MGLL* knockdown reduced PA levels, whereas *MGLL* overexpression increased them (Fig. 4K). Together, these findings indicate that MGLL regulates both glycerolipid metabolism and endothelial cell senescence, underscoring its potential as a key driver of vascular aging.

### Identification of MGLL as a novel target for TZ intervention in vascular endothelial cell senescence

**Fig. 5:**
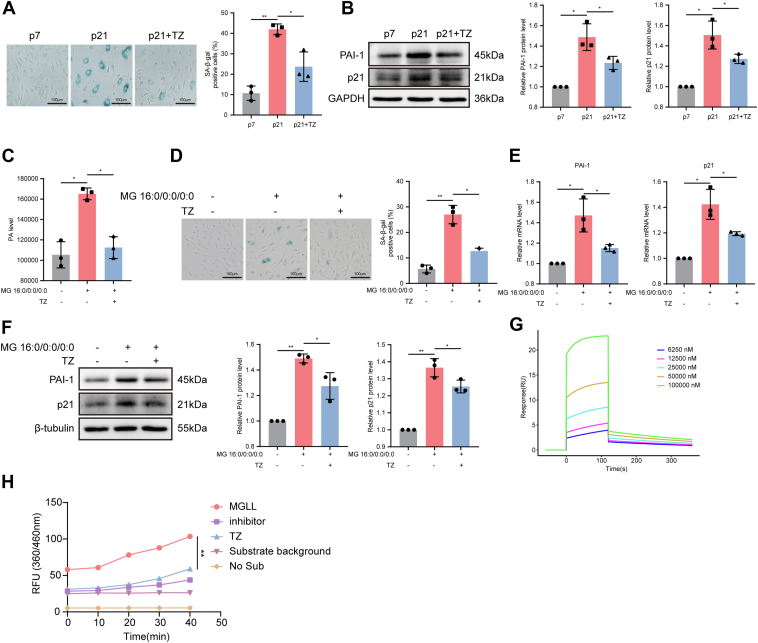
TZ delays vascular endothelial cell senescence by inhibiting MGLL enzymatic activity. A: Representative images and quantification of SA-β-gal staining in HUVECs (n = 3). B: Representative Western blots and quantification of p21 and PAI-1 protein expression levels in HUVECs at p7, p21, and p21 + TZ (n = 3). C: Quantification of intracellular PA levels in HUVECs at p7, p21, and p21 + TZ using GC-MS-based targeted FFA lipidomics (n = 3). D–F: Representative images and quantification of SA-β-gal staining (D), relative mRNA levels of *p21* and *PAI-1* by RT-qPCR (E), and representative Western blots with quantification of p21 and PAI-1 protein expression levels (F) in HUVECs treated with MG 16:0/0:0/0:0 ± TZ (n = 3). G: SPR sensorgrams showing the interaction between different concentrations of TZ and human MGLL protein. H: Reaction kinetics of TZ- and MGLL-specific inhibitors on the activity of MGLL (n = 3). Error bars represent the mean ± standard deviation. Statistical analyses: one-way ANOVA (A and B, D-F), paired *t*-test (C), and two-way ANOVA (H). ∗*P* < 0.05, ∗∗*P* < 0.01.

In the replicative senescence model, TZ significantly reduced senescence, as evidenced by SA-β-gal staining and WB analysis (Fig. 5A, B). To further investigate its impact on PA regulation, we assessed changes in intracellular FFA levels following MG 16:0/0:0/0:0 stimulation. Intracellular PA levels were quantified using a targeted FFA lipidomics approach based on GC-MS. The results revealed that exposure to MG 16:0/0:0/0:0 led to an increase in PA levels, which was mitigated by TZ treatment (Fig. 5C). Subsequently, we explored the effects of TZ on MG 16:0/0:0/0:0-induced senescence in HUVECs. SA-β-gal staining indicated that TZ ameliorated MG 16:0/0:0/0:0-induced senescence (Fig. 5D). RT-qPCR and WB experiments further demonstrated that TZ reduced the expression of p21 and PAI-1 induced by MG 16:0/0:0/0:0 (Fig. 5E, F). These findings suggest that TZ may inhibit endothelial cell senescence, at least in part, by reducing intracellular PA accumulation.

MGLL is known to selectively hydrolyze MAGs, rather than triacylglycerols (TAGs) or DGs, producing fatty acids and glycerol (51). This specificity indicates that TZ's regulation of PA levels under MG 16:0/0:0/0:0 stimulation is likely mediated through targeting MGLL. SPR analysis demonstrated that the response signal intensity between MGLL and TZ increased with TZ concentration, yielding a binding constant (KD) of 0.723 mM, indicative of a measurable binding affinity between the two molecules (Fig. 5G). Additionally, enzyme activity assays confirmed that TZ inhibited MGLL activity (Fig. 5H). These results suggest that TZ's antisenescence effects are, at least in part, mediated through the inhibition of MGLL, establishing MGLL as a potential therapeutic target for vascular aging.

### Key amino acid residues in the interaction of TZ and MGLL

**Fig. 6:**
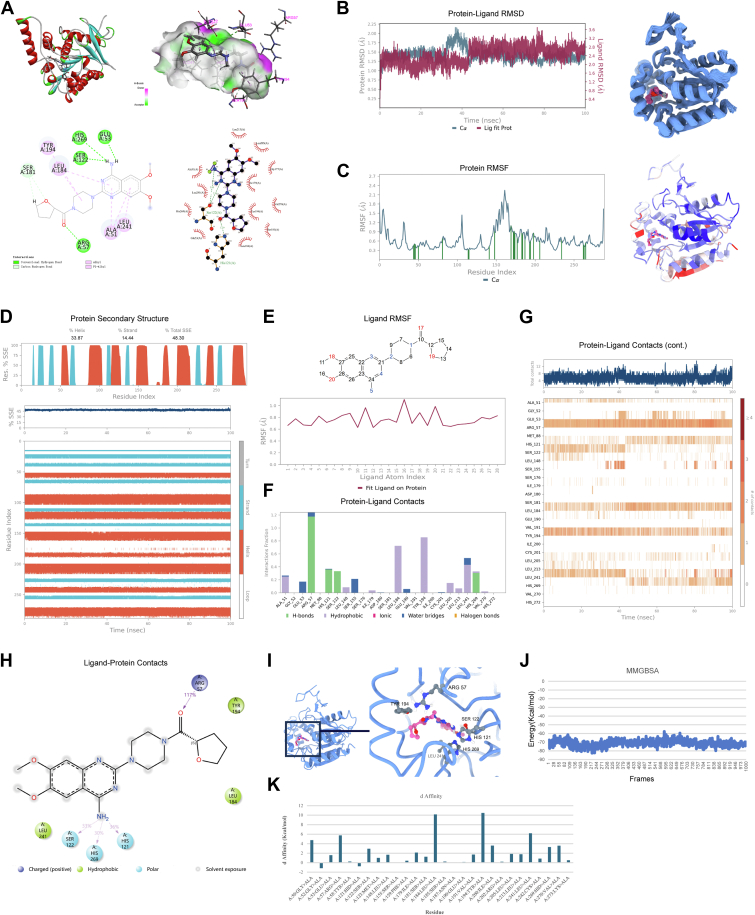
Key amino acid residues mediating TZ-MGLL binding. A: Molecular docking prediction of TZ binding to MGLL. B: MD simulation showing the stable binding mode of TZ to MGLL. RMSD curves for MGLL (blue) and TZ (red) over a 100 ns MD simulation, with 100 conformational snapshots saved per 1 ns. C: RMSF of MGLL during 40–100 ns range of the MD simulation (the green marked part represents the location of amino acids interacting with the molecule). B-factor distribution map, where red regions indicate areas of higher flexibility and blue regions indicate less flexibility. D: Secondary structure distribution of MGLL throughout the MD simulation, illustrating changes in secondary structure content and conformational transitions over the 100 ns simulation. E: RMSF of TZ during 40–100 ns MD simulation. F: Contribution analysis of key amino acids at the binding sites for TZ binding. G: Time-resolved analysis of the total interactions between TZ and MGLL throughout the MD simulation, including interactions of key amino acids with TZ over time. H: Two-dimensional representation of TZ-MGLL binding patterns from the MD simulation. I: Three-dimensional depiction of the binding mode between TZ and MGLL following MD optimization. J: Molecular mechanics/generalized born surface area binding free energy of the TZ-MGLL complex over the last 10 ns of the MD trajectory (1,000 frames, each frame calculated individually). K: Alanine scanning of residues within 5 Å of the binding site.

To elucidate the interaction mechanism between TZ and MGLL, we investigated the key amino acid residues involved in binding. Molecular docking analysis identified Ala51, Glu53, Arg57, His121, Ser122, Ser181, Tyr194, Leu213, and His269 as critical residues within the binding pocket (Fig. 6A). Subsequently, MD simulations were performed to assess the stability of the TZ-MGLL interaction. Root mean square deviation values of both MGLL and TZ reached stability after ∼40 ns, and the 40–100 ns trajectory was selected for further sampling and analysis. The superimposed conformations from this interval demonstrated strong structural consistency, confirming that both the protein and the small molecule maintained a stable interaction throughout the simulation (Fig. 6B). RMSF and B-factor analyses showed that MGLL's overall structure exhibited low flexibility, with RMSF values below 2.0 Å in most regions, highlighting the structural stability of MGLL during the MD simulation (Fig. 6C). Analysis of MGLL's secondary structure further supported this stability, showing predominantly α-helix content without significant conformational changes (Fig. 6D). Moreover, RMSF analysis of TZ revealed that most atoms remained in a stable configuration, underscoring TZ's consistent binding at the MGLL active site (Fig. 6E).

Detailed interaction analysis within the stable 40–100 ns interval revealed specific residues—Arg57, Leu184, Tyr194, Leu241, His121, Ser122, and His269—that played essential roles in strengthening the binding through hydrogen bonding and hydrophobic interactions (Fig. 6F). The number of contacts between TZ and MGLL remained stable at 4–8, indicating a robust binding interaction (Fig. 6G). Statistical analysis of interactions over the 40–100 ns trajectory further showed that Arg57, Ser122, His121, and His269 frequently formed hydrogen bonds with TZ, contributing significantly to the binding stability (Fig. 6H). After MD optimization, the three-dimensional configuration illustrated the spatial arrangement of key residues, including Arg57, His121, Ser122, and His269, around TZ (Fig. 6I). The binding free energy (molecular mechanics/generalized born surface area) of the TZ-MGLL complex remained stable at approximately −71.40 kcal/mol, consistent with strong binding affinity (Fig. 6J). Alanine scanning mutagenesis revealed significant shifts in binding free energy upon mutation of the key hydrogen-bonding residues, Arg57, His121, Ser122, and His269 (Fig. 6K).

In conclusion, Arg57, His121, Ser122, and His269 are critical residues for TZ binding to MGLL. Notably, Ser122 and His269 are known to be components of the enzyme catalytic center of MGLL (52, 53), suggesting that TZ may bind directly with the enzyme’s active site to modulate its activity.

## Discussion

Vascular aging, driven by endothelial dysfunction and accelerated by cellular senescence—which is marked by increased oxidative stress, chromatin remodeling, and metabolic reprogramming—is a major contributor to CVDs, the leading cause of global mortality (4, 54, 55). Lipid metabolic dysfunction, particularly the accumulation of FFAs, such as PA, has emerged as a key driver of endothelial senescence (56, 57, 58, 59, 60, 61, 62, 63, 64). Understanding these mechanisms is therefore essential for identifying novel therapeutic targets to combat age-related CVDs.

This study provides novel evidence that TZ, an α_1_-adrenergic receptor antagonist primarily employed in the treatment of BPH and hypertension (65, 66), alleviates age-related endothelial dysfunction by inhibiting MGLL-mediated PA accumulation. This study identifies MGLL as a key regulator of PA accumulation and endothelial dysfunction, offering a promising therapeutic target for vascular aging. Collectively, our findings provide new mechanistic insight into TZ’s action and lay the groundwork for future clinical applications of TZ in treating age-related vascular diseases.

Clinically, TZ treatment improved endothelial function, as indicated by increased FMD in patients. Consistent with the clinical findings, aged mice treated with TZ exhibited reduced vascular stiffness, improved endothelium-dependent vasodilation, and diminished expression of senescence markers. Nevertheless, the precise molecular mechanisms underlying TZ's beneficial effects on endothelial senescence and function remain elusive.

Previous research has suggested that TZ enhances lipid metabolism (67). Proteomic analyses conducted on replicative senescence models have further emphasized the significant role of lipid metabolism in endothelial aging. This raised the intriguing possibility of a connection between TZ’s lipid metabolic improvements and its impact on endothelial senescence. Considering the established ability of TZ to enhance lipid metabolism (68), we postulated that these effects might contribute to its antisenescence properties. In alignment with this hypothesis, our lipidomic analysis revealed that TZ modulates glycerolipid metabolism by decreasing the accumulation of PA, a key driver of endothelial senescence. Both PA and MG 16:0/0:0/0:0, which are crucial intermediates in glycerolipid metabolism, were identified as potent inducers of senescence in HUVECs. Moreover, TZ treatment lowered plasma PA levels in patients, further supporting its potential therapeutic value in mitigating vascular aging.

To further elucidate the key enzymatic targets through which TZ regulates the glycerolipid metabolic pathway, we analyzed lipid metabolic enzymes altered in replicative aging and crossreferenced them with known TZ targets. This analysis identified MGLL and FASN as key candidates. Interestingly, although FASN, a critical enzyme in de novo PA biosynthesis, was downregulated in senescent endothelial cells, PA levels were paradoxically elevated. This finding highlights MGLL-mediated MAG hydrolysis, rather than de novo synthesis, as the predominant source of PA accumulation in senescent cells. Bioinformatic analysis utilizing the HPA and UniProt databases revealed that *MGLL* was abundantly expressed in endothelial cells. Indeed, we confirmed that MGLL expression was upregulated in both senescent cells and the aortic endothelium. Functional studies demonstrated that *MGLL* knockdown attenuated, whereas *MGLL* overexpression exacerbated, senescence phenotypes, thus positioning *MGLL* as a functional regulator of vascular aging. Although MGLL expression correlated with classical senescence markers (p21, PAI-1, and SA-β-gal), it has not yet been validated as a definitive senescence biomarker in terms of sensitivity or specificity. We therefore propose MGLL as a candidate metabolic marker of endothelial senescence, which reflects the underlying senescence-associated metabolic remodeling.

This study investigated the impact of TZ on vascular aging and glycerolipid metabolism utilizing an in vitro model based on HUVECs. Within the context of glycerolipid metabolism, MGLL catalyzes the hydrolysis of MAG into FFAs and glycerol (49). Our experimental findings demonstrated that cells exposed to MG 16:0/0:0/0:0 exhibited elevated PA levels. Notably, treatment with TZ mitigated this increase, suggesting that TZ's influence on PA levels is mediated through the inhibition of MGLL. Molecular docking simulations and SPR experiments substantiated the direct binding of TZ to MGLL, further validating MGLL as a potential therapeutic target. MD simulations identified Ser122 and His269, which are part of MGLL’s catalytic triad (52, 69), thereby providing a structural explanation for its inhibitory effect.

The therapeutic significance of MGLL transcends its role in endothelial aging. Its expansive binding pocket enables the accommodation of diverse ligands, such as naturally occurring fatty acids, glycerol, and synthetic compounds like piperidine-carbamate JZL184 (51, 52, 69). Notably, MGLL exhibits selectivity for MAG hydrolysis, distinguishing it from triacylglycerol and DG, making it a promising drug target (70). Our work pioneers the identification of MGLL as a regulator of endothelial senescence and a direct molecular target of TZ, thereby expanding TZ’s therapeutic scope beyond BPH to vascular and metabolic aging.

Several limitations warrant consideration. First, our mechanistic investigations were performed primarily in a replicative senescence model of HUVECs, which closely mimics gradual age-related endothelial changes but may not capture all features of stress-induced senescence (e.g., oxidative stress, hyperglycemia, or inflammation). Second, although other metabolic pathways such as de novo lipogenesis or exogenous fatty acid uptake may also contribute to intracellular PA pools, our data—together with reduced FASN expression in senescent ECs—support MGLL-mediated MAG hydrolysis as the predominant source under our experimental conditions. Third, our functional validation focused exclusively on MG 16:0/0:0/0:0 (1-monopalmitin) and did not investigate other palmitoyl-MAG isomers (e.g., 2-monopalmitin), which may also contribute to palmitate accumulation. In addition, glycerol—produced from the hydrolysis of all MAG species—is not specific for palmitoyl-MAG hydrolysis and thus cannot be used alone to infer MGLL substrate specificity. Fourth, we focused on intracellular PA levels but not extracellular secretion or systemic levels, which would provide broader physiological context. Fifth, regarding target selectivity, TZ is a known ligand for α_1_-adrenergic receptors and phosphoglycerate kinase 1; we did not perform SPR assays against unrelated control proteins and therefore cannot fully exclude off-target binding. Finally, our in vivo design did not include a “young + TZ” group, which limits conclusions regarding age dependency of TZ’s effects. Taken together, these limitations should be addressed in future studies to further strengthen the mechanistic understanding and translational relevance of targeting MGLL in vascular aging.

In conclusion, this study identifies MGLL as a novel regulator of endothelial senescence and a direct target of TZ. By inhibiting MGLL-mediated PA accumulation, TZ alleviates endothelial dysfunction and vascular aging. These findings highlight MGLL as a promising therapeutic target and extend the potential clinical utility of TZ to age-related CVDs.

## Data availability

Lipidomic and FFA datasets generated in this study are available in supplemental Table S1 (Raw lipidomic data) and Supplemental FFAs Raw Intensities, respectively. All other data supporting the findings of this study are available from the corresponding author upon reasonable request (Cuntai Zhang, Department of Geriatrics, Tongji Hospital, Tongji Medical College, Huazhong University of Science and Technology; email: ctzhang@tjh.tjmu.edu.cn).

### Supplemental data

This article contains supplemental data.

### Ethics approval and consent to participate

The protocol of the present study, involving animal experimentation, was approved by the Ethics Committee of Tongji Hospital, Huazhong University of Science and Technology.

### Patient consent for publication

Not applicable.

## Conflict of interest

The authors declare that they have no conflicts of interest with the contents of this article.

## Supplemental Data

Supplementary Tables S2 and S3

Supplementary FFAs Raw Intensities
