# Supplementary material for: Targeting MGLL: terazosin regulates glycerolipid metabolism to mitigate endothelial cell senescence
Source: J Lipid Res. 2025 Sep 17;66(11):100904. doi: 10.1016/j.jlr.2025.100904 (PMC12589924; doi:10.1016/j.jlr.2025.100904)
Supplement: Supplementary Tables S2 and S3 [file mmc2.pdf]

Table S2

Table S2 Analysis of the contents of CARs, TGs, and DGs

CARs

Index	Compounds	p5-1	p5-2	p5-3	p21-1	p21-2	p21-3	TZ-1	TZ-2	TZ-3	TZ_vs_p21_VIP	TZ_vs_p21_Fold_Change	TZ_vs_p21_Type	p21_vs_p5_VIP	p21_vs_p5_Fold_Change	p21_vs_p5_Type
LIPID-P-0044	Carnitine C22:1	0.6552076	0.7596981	0.6622280	0.8537960	1.0641772	1.1256511	0.7117720	1.0469861	0.9206550	--	--	--	1.11018502207844	1.465300171393247	up
LIPID-P-0042	Carnitine C18:1	0.0285641	0.0375764	0.0434412	0.1897324	0.2340756	0.5882162	1.5522883	0.2823333	0.4704076	1.1927017554003	2.277642246065433	up	--	--	--
LIPID-P-0035	Carnitine C12:1	0.1869918	0.1727117	0.1753284	0.2828087	0.3164355	0.3300781	0.2905942	0.2784120	0.4054466	--	--	--	1.25252776290284	1.7369473887753668	up
LIPID-P-0023	Carnitine C18:0	0.1855194	0.1517062	0.1976633	0.7088117	0.7455742	0.7596029	1.0906480	0.6666204	0.8624140	--	--	--	1.28089393225807	4.139155826916815	up
LIPID-P-0021	Carnitine C16:0	0.3813455	0.3582601	0.4422298	1.5447085	1.8726051	2.3697919	6.1429418	1.6351806	2.3296380	1.1809306623325	1.746600315106014	up	1.22223356727082	4.896709991774278	up
LIPID-P-0011	Carnitine C6-2OH	0.9423210	0.7060174	0.6376596	1.1240752	1.1855498	1.2758790	1.1219145	1.1607037	1.3395418	--	--	--	1.17352157756227	1.5684632261598723	up
LIPID-P-0003	Carnitine C3:0	0.1102810	0.0784204	0.0866591	0.1448052	0.1447801	0.1961426	0.1949556	0.1882222	0.1944351	--	--	--	1.13061983966985	1.7639701671965504	up
LIPID-P-0028	Carnitine C5:1	0.0976185	0.1031602	0.0741516	0.1385404	0.1207223	0.1307617	0.1109040	0.1625377	0.1986912	1.0617304719646	1.210521167935651	up	1.13619760678767	1.4186297880881384	up

TGs

Index	Compounds	p5-1	p5-2	p5-3	p21-1	p21-2	p21-3	TZ-1	TZ-2	TZ-3	TZ_vs_p21_VIP	TZ_vs_p21_Fold_Change	TZ_vs_p21_Type	p21_vs_p5_VIP	p21_vs_p5_Fold_Change	p21_vs_p5_Type
LIPID-P-2327	TG(18:0_16:1_20:1)	696.43415	374.59769	584.05607	1002.3601	1077.1814	1309.7332	1167.8946	833.27552	1538.9051	--	--	--	1.17831211433865	2.047791383004559	up
LIPID-P-2338	TG(16:0_22:1_18:2)	32.539524	17.154473	28.365247	38.841454	48.115549	45.914719	42.485615	37.448382	67.201097	--	--	--	1.11857109274345	1.7021907252402724	up
LIPID-P-2286	TG(14:0_18:0_18:1)	83.042043	45.861961	73.481624	108.46968	129.60855	177.73439	143.64184	100.58125	174.05084	--	--	--	1.08057392202674	2.054556125704228	up
LIPID-P-2325	TG(18:0_17:1_18:1)	56.980976	33.608765	42.324522	76.250965	94.280468	91.406262	70.809359	53.721763	87.809433	1.5132198671738	0.8106529161275654	down	1.19612354735534	1.970726749268635	up
LIPID-P-1020	TG(24:0_18:1_18:2)	6.3459434	3.6992981	4.3441264	9.3076295	10.099930	10.537110	10.115622	7.8229867	13.440219	--	--	--	1.22751264691332	2.081027539627589	up
LIPID-P-1171	TG(16:0_16:1_22:6)	1.7226806	2.5556665	3.2720540	4.0452389	6.0469542	4.7395839	6.1797259	4.0781484	4.8608793	--	--	--	1.07930217819901	1.9643693807135132	up
LIPID-P-1177	TG(16:0_18:1_22:6)	6.0661918	3.9443620	6.8679633	17.022222	14.239601	9.0771496	17.821888	12.352084	15.501053	--	--	--	1.09239878060831	2.3899595778729044	up
LIPID-P-1181	TG(18:0_18:1_22:6)	2.1054986	3.3725462	3.3837282	4.1884332	7.8458689	8.1461599	8.4235550	7.9602321	10.931378	1.3994592943936	1.3535450992897826	up	1.02908241127048	2.277248779740856	up
LIPID-P-1206	TG(18:0_18:2_22:6)	1.8110232	1.8088050	5.3268594	6.1036570	7.4123954	9.5849622	17.969024	10.195371	7.5713236	1.3947701086644	1.5469328838181422	up	1.06665727307797	2.582074545365941	up
LIPID-P-1010	TG(18:1_18:2_22:0)	26.355542	14.587137	20.213030	33.292674	45.731445	44.433599	35.312719	30.586113	55.776910	--	--	--	1.15386446613549	2.018743950937428	up
LIPID-P-1009	TG(18:1_18:1_22:1)	1.6637856	1.8321445	1.3289229	2.8996845	4.5297972	3.3430994	2.3909654	1.7528195	4.9280804	--	--	--	1.13273997257328	2.232727289641212	up
LIPID-P-0999	TG(18:1_18:1_20:1)	50.208044	28.357395	40.426060	90.570395	70.439430	96.695976	78.717937	73.916441	87.809433	--	--	--	1.19102669542108	2.1657496609324407	up
LIPID-P-0997	TG(18:1_18:2_20:0)	30.036483	13.536863	25.461717	45.643183	34.461136	46.337896	39.358968	35.487734	49.952815	--	--	--	1.09816005506676	1.8315651036906893	up
LIPID-P-0991	TG(16:0_18:1_20:2)	83.042043	38.393346	62.090855	95.582195	98.398466	136.89780	98.581342	84.896071	117.37791	--	--	--	1.04167896705815	1.802894538924036	up
LIPID-P-0988	TG(18:1_18:1_18:1)	1187.7772	462.92281	871.10343	1264.9471	1567.8083	1424.7686	1385.6012	1091.4831	1941.4621	--	--	--	1.02915240817134	1.688285406346721	up
LIPID-P-0984	TG(17:1_18:1_18:1)	28.196013	17.621262	23.842441	34.008646	31.860296	37.027999	40.370531	39.409030	59.360969	1.5855264602927	1.3522319318186935	up	1.11474016455084	1.4771369501865286	up
LIPID-P-0981	TG(16:1_18:1_18:1)	197.29847	122.53195	182.02894	259.53966	381.45660	332.19405	349.44878	348.99539	501.76819	1.1943550633197	1.2332761002428845	up	1.13328369148279	1.939169372807595	up
LIPID-P-1169	TG(18:2_18:2_18:3)	168.58712	95.108138	128.98370	275.64902	269.83720	293.05016	234.49852	223.51390	389.76636	--	--	--	1.24183649862343	2.135424787661563	up
LIPID-P-1044	TG(16:1_18:1_18:2)	1192.6250	610.32584	655.52756	1541.1286	1322.0939	1379.5574	1736.2087	1535.1876	1845.7901	1.9616607940880	1.2060927976209164	up	1.09427545006007	1.725774730007422	up
LIPID-P-1046	TG(16:0_16:0_20:4)	4.2846160	3.4659039	2.8253572	5.8530670	9.7748256	6.8554696	5.4256522	6.7838431	11.132981	--	--	--	1.08208948928273	2.1259099071620278	up
LIPID-P-1048	TG(16:0_18:2_18:2)	672.87613	408.43985	546.08684	1208.2019	1595.1821	988.11862	892.01401	1131.2940	1209.6197	--	--	--	1.13466574070842	2.329787461875523	up
LIPID-P-1053	TG(18:1_18:1_18:2)	1023.3017	507.63239	854.30763	1360.3458	1527.9938	1502.2788	1517.3434	1284.2246	2385.6389	--	--	--	1.17157670961472	1.84074356185356	up
LIPID-P-1056	TG(16:0_18:0_20:4)	340.11900	149.37229	188.72940	386.62461	494.15969	603.02742	343.93117	409.77549	425.60694	1.5750475468960	0.7947865581394703	down	1.09506584054959	2.187800721776998	up
LIPID-P-1060	TG(16:0_18:1_22:3)	65.226286	36.176101	63.542620	100.05701	73.690481	85.481782	68.970155	73.916441	111.10581	--	--	--	1.04155568875383	1.5716103453849244	up
LIPID-P-1096	TG(16:0_18:2_18:3)	110.57548	47.962508	67.674565	165.21042	107.06793	153.82489	143.09008	127.05000	143.13833	--	--	--	1.04472828617253	1.8836409687499776	up
LIPID-P-1097	TG(16:1_18:1_18:3)	100.71056	54.380849	47.126512	151.24898	112.48635	139.43686	141.80264	112.54121	157.02656	--	--	--	1.12011165397907	1.9937510396770848	up
LIPID-P-1105	TG(16:0_18:1_20:4)	13.030533	10.607766	18.091220	21.479145	33.377453	29.833988	16.368916	25.096298	15.254649	1.7670798860461	0.6697304443479348	down	1.10334820501567	2.029512574187775	up
LIPID-P-0973	TG(16:1_16:1_18:1)	127.65505	56.714791	67.116194	180.78280	163.85295	126.31837	106.48992	71.171533	148.73842	1.6174054979309	0.6930608722069355	down	1.05168930639758	1.8726849736489817	up
LIPID-P-0965	TG(14:1_16:1_18:1)	9.7176859	7.6203207	10.910569	24.343031	27.092088	13.943686	15.706803	11.273727	30.016490	--	--	--	1.07010240848822	2.314410688171853	up
LIPID-P-0856	TG(18:0_18:1_20:0)	8.5839559	5.3563969	6.3765968	12.117817	12.527381	10.325522	13.315838	9.7052090	12.297800	--	--	--	1.17317329799487	1.7212584611850181	up
LIPID-P-0852	TG(18:0_18:0_18:1)	164.90618	91.607225	130.65881	210.31662	224.32249	326.90434	227.14171	212.73034	383.04625	--	--	--	1.07227135129299	1.9669372245466432	up
LIPID-P-0851	TG(16:0_18:0_20:1)	38.134555	20.655387	31.380450	46.538147	52.450283	64.534513	45.796183	49.212272	82.657349	--	--	--	1.11276795407562	1.8134882166294384	up
LIPID-P-0850	TG(17:0_17:0_19:1)	3.5778752	2.0538689	3.0598731	4.9581026	5.0716390	6.2418628	7.3200325	5.8427319	7.6833254	1.7854446979341	1.281133028439496	up	1.16966324889628	1.8721031715115213	up
LIPID-P-0849	TG(17:0_18:0_18:1)	14.635423	5.6014608	8.4090673	17.899287	16.363621	25.813805	19.311643	15.508728	19.824323	--	--	--	1.04280964158860	2.0972147896843487	up
LIPID-P-0847	TG(16:0_18:0_18:1)	1304.5257	940.57864	1373.5926	2577.4974	2514.1458	2983.3988	2648.4539	1799.8751	3158.4515	--	--	--	1.24551563654990	2.2314777940372195	up
LIPID-P-0838	TG(14:0_16:0_18:1)	67.140375	51.113330	70.131398	124.57904	130.04202	165.25067	111.45577	129.40278	101.69766	1.6327333337165	0.8158591948604523	down	1.20952219332592	2.228794793719405	up
LIPID-P-0813	TG(16:0_18:0_24:0)	0.8068624	1.4820531	1.2619184	2.4880009	2.3841038	3.3430994	3.6048401	2.6076622	3.8304625	1.2755800957828	1.2224851265011552	up	1.15328725425815	2.313598440598948	up
LIPID-P-0810	TG(16:0_16:0_24:0)	2.6944492	1.7037776	2.3339908	4.6538147	4.9632706	5.4801439	4.1198173	4.7447688	5.7792943	--	--	--	1.24638701925366	2.2425343146389416	up
LIPID-P-0809	TG(16:0_18:0_22:0)	6.3017721	3.6992981	4.4222983	9.0570395	8.8862051	9.8600273	10.998440	9.7052090	9.9905631	--	--	--	1.21758577357085	1.927654540731496	up
LIPID-P-0804	TG(18:0_18:0_18:0)	125.64268	84.021913	96.039813	164.07695	164.71989	220.75723	252.58388	190.18288	214.29701	--	--	--	1.13451006053833	1.7976648731516365	up
LIPID-P-0800	TG(16:0_18:0_18:0)	462.32627	383.93346	664.46149	831.42190	778.08478	1001.8718	1094.3264	842.09844	1332.8217	1.3597369275201	1.2519236817759538	up	1.11093485957469	1.7285641562806888	up
LIPID-P-0798	TG(14:0_18:0_20:0)	8.9373263	6.0682493	10.184687	14.033041	16.645379	17.900393	11.053616	13.567686	22.041959	--	--	--	1.18343144414827	1.928475864922521	up
LIPID-P-0858	TG(16:0_18:1_22:0)	17.815757	9.4641349	12.619184	24.880009	22.107144	33.430994	32.186072	21.175001	27.328446	--	--	--	1.10621399755386	2.015539046414544	up
LIPID-P-0867	TG(16:0_20:1_22:0)	3.1214385	1.2603287	1.8984614	4.2421311	3.7278714	3.7027999	3.7519764	3.3723150	4.0768665	--	--	--	1.10787981628021	1.8586588497648524	up
LIPID-P-0939	TG(22:0_18:1_18:1)	7.7447012	4.0143803	4.7238187	15.948265	11.140266	14.768882	16.019468	9.1170146	12.835409	--	--	--	1.19372793499438	2.539444738004837	up
LIPID-P-0934	TG(18:0_18:2_20:0)	17.374044	16.104200	15.746062	32.218717	36.411767	41.048182	35.864481	34.507410	44.128720	--	--	--	1.24234431549311	2.2281404065496044	up
LIPID-P-0933	TG(18:0_18:1_20:1)	21.643936	16.804382	21.776469	31.323753	31.210086	34.912114	32.553913	29.213659	49.952815	--	--	--	1.22801920676597	1.618037280648874	up
LIPID-P-0925	TG(16:1_18:0_20:1)	157.54430	68.034410	118.37465	220.16123	260.08405	275.06514	228.06131	190.96714	257.60420	--	--	--	1.18355954861260	2.1959675468741318	up
LIPID-P-0923	TG(18:0_18:1_18:1)	385.02649	166.29337	284.21084	440.32247	460.56550	617.83862	463.47944	404.87387	523.04854	--	--	--	1.04891811404126	1.8176789805892228	up
LIPID-P-0920	TG(16:0_16:1_20:1)	17.079569	11.412976	13.624252	20.226194	21.890407	30.680342	27.220221	14.293126	30.240493	--	--	--	1.05554153622420	1.72845392132447	up
LIPID-P-0915	TG(16:0_18:1_18:1)	5057.6138	2316.4374	3232.9681	6345.2974	7531.6006	5437.8262	5158.9676	6293.6810	8736.1426	--	--	--	1.07091692567668	1.8209379650192872	up
LIPID-P-0911	TG(14:0_18:0_18:2)	133.10284	60.682493	99.725061	130.12782	187.04378	170.54038	116.78946	109.20811	179.42692	1.1788559461101	0.8312785277740872	down	1.00071957363672	1.6616514470550432	up
LIPID-P-0901	TG(16:0_16:1_16:1)	67.876563	31.624914	41.989499	62.468513	93.196785	95.638033	81.476744	65.877782	86.465411	--	--	--	1.00051472798260	1.7761085234061298	up
LIPID-P-0894	TG(14:0_14:1_18:1)	35.925990	20.888781	18.537917	66.943335	72.606797	46.972662	58.670612	26.468752	46.368757	1.4236649301688	0.7050512033746917	down	1.15170228030341	2.4753303171695324	up
LIPID-P-0884	TG(14:0_14:1_16:1)	15.459954	6.6050559	12.954207	22.732095	22.107144	31.315108	25.197096	24.704168	31.584515	--	--	--	1.12449338383593	2.1746444327522525	up
LIPID-P-0916	TG(16:0_18:0_18:2)	541.83461	184.38142	399.79364	719.55136	823.59949	1227.2137	763.26972	760.73154	1070.7374	--	--	--	1.07241018121565	2.4603381589291797	up
LIPID-P-0806	TG(16:0_18:0_20:0)	21.791174	11.786407	11.725791	22.553102	35.328083	28.352868	28.691584	25.096298	34.272559	--	--	--	1.04418258544635	1.9034797799849013	up

DGs

Index	Compounds	p5-1	p5-2	p5-3	p21-1	p21-2	p21-3	TZ-1	TZ-2	TZ-3	TZ_vs_p21_VIP	TZ_vs_p21_Fold_Change	TZ_vs_p21_Type	p21_vs_p5_VIP	p21_vs_p5_Fold_Change	p21_vs_p5_Type
LIPID-P-0266	DG(18:0_22:6)	1310.4152	729.35688	904.56103	1154.5040	1235.3992	997.64010	677.74673	946.01280	759.37239	2.0836531998008	0.7034985688577942	down	--	--	--
LIPID-P-0261	DG(16:0_22:6)	1236.7963	665.17348	865.47505	775.03915	1972.3040	1354.1668	937.99411	1048.9468	1220.8199	1.0184392425449	0.7820926553817512	down	--	--	--
LIPID-P-0218	DG(18:1_18:1)	14429.291	11932.278	13400.904	4922.3040	6122.8120	3475.3422	3572.6540	5097.6855	4625.6755	--	--	--	1.23970185906392	0.365179952607716	down
LIPID-P-0201	DG(18:0_18:1)	3747.1985	2602.3453	1859.3754	1208.2019	1300.4202	988.11862	1011.5622	2166.5163	991.21618	--	--	--	1.02948614038251	0.42596846345388895	down
LIPID-P-0195	DG(16:1_18:0)	1590.1667	1277.8332	1446.1809	532.50380	562.43176	442.22011	453.36382	589.17481	208.32340	--	--	--	1.24846943083592	0.3563030136056002	down
LIPID-P-0191	DG(14:0_18:1)	612.50869	939.41167	764.96827	450.16708	609.03015	454.91542	506.70074	401.93290	710.09159	--	--	--	1.02853867783004	0.6535111946935446	down
LIPID-P-0174	DG(16:0_18:0)	14282.053	14587.137	14015.112	15124.898	19289.567	16398.114	23449.852	20782.872	17136.279	1.5906143392518	1.2077522051791336	up	--	--	--
LIPID-P-0171	DG(12:0_20:0)	339.38281	245.64739	236.74930	417.05340	393.37712	351.23702	562.79647	529.37504	359.52587	1.5521457322883	1.249666805775956	up	1.08739121352860	1.4136000205990742	up
LIPID-P-0170	DG(14:0_18:0)	1126.3681	805.21000	647.71036	975.51117	882.11841	714.11142	648.31946	872.48849	592.48967	1.3147647573682	0.8217381252857204	down	--	--	--
LIPID-P-0196	DG(16:0_18:1)	25545.735	25556.665	22669.862	11097.558	13871.149	10547.690	9471.9013	13430.440	7145.7166	--	--	--	1.25505252206703	0.4814329450573847	down

Table S3

Table S3 Analysis of the contents of palmitate-containing PCs and PEs

PEs

Index	Compounds	p5-1	p5-2	p5-3	p21-1	p21-2	p21-3	TZ-1	TZ-2	TZ-3	TZ_vs_p21_VIP	TZ_vs_p21_Fold_Change	TZ_vs_p21_Type	p21_vs_p5_VIP	p21_vs_p5_Fold_Change	p21_vs_p5_Type
LIPID-N-0203	PC(16:0_14:0)	5440.4317	5893.2036	4081.6920	6291.5995	7694.1532	7246.9085	6565.9588	7136.7598	7459.3218	--	--	--	1.04057450732797	1.3773733551804113	up
LIPID-N-0291	PC(16:0_22:5)	161.22524	175.62913	126.75021	332.92674	353.28083	319.49874	337.49396	328.40859	381.92623	--	--	--	1.25831393647401	2.1693191232870617	up
LIPID-N-0284	PC(16:0_20:5)	42.551685	49.421222	35.121536	73.566071	92.546575	92.252616	87.362197	94.895377	98.449607	--	--	--	1.20660170473405	2.032860403361028	up
LIPID-N-0272	PC(16:0_20:4)	839.25469	921.90710	675.62891	1485.6408	1527.9938	1576.3348	1535.7354	1656.7478	1960.0320	--	--	--	1.25307445115102	1.8836125253906368	up
LIPID-N-0303	PC(16:0_22:6)	298.15627	336.08765	237.86604	491.33544	548.34387	463.37896	532.44960	542.11925	702.25146	--	--	--	1.20210970943509	1.723473334759834	up
LIPID-N-0309	PC(16:0_24:6)	17.226806	16.279245	8.2638908	33.919149	42.372026	44.856776	39.358968	38.428706	46.480758	--	--	--	1.21779466131882	2.900361919019247	up
LIPID-N-0212	PC(16:0_24:0)	35.484277	40.260500	31.547961	52.444912	58.627280	60.408536	53.612801	56.858800	60.256983	--	--	--	1.21318821634091	1.5982510104412255	up
LIPID-N-0208	PC(16:0_18:0)	3813.4555	4014.3803	3059.8731	4573.2679	4876.5759	4940.5931	4754.3427	4529.0975	5409.6883	--	--	--	1.12887864295921	1.3217139710143473	up
LIPID-N-0206	PC(16:0_16:0)	7545.9303	7964.5772	5834.9770	9397.1259	10891.019	8992.5142	9012.1003	10587.500	11536.188	--	--	--	1.06671816871127	1.3717495957402086	up
LIPID-N-1013	PC(16:0_17:0)	111.16443	126.61635	74.821714	282.80874	317.51928	316.32491	274.96102	301.93983	259.84424	--	--	--	1.25841297950848	2.932327513764746	up
LIPID-N-1014	PC(16:0_17:1)	40.269501	33.550416	28.867781	83.589673	70.764535	70.353199	60.233936	124.50116	90.721481	--	--	--	1.24862325883427	2.188260224420838	up
LIPID-N-0221	PC(16:0_20:1)	949.68294	1032.7693	787.30311	1208.2019	1267.9097	1269.5314	1186.2866	1303.8311	1355.2221	--	--	--	1.16114040964897	1.3523371276319682	up
LIPID-N-0223	PC(16:0_22:1)	77.299774	81.687971	63.095923	133.34969	138.71149	112.14194	140.69911	133.32408	147.84241	--	--	--	1.20469935365258	1.7299927068918135	up
LIPID-N-0211	PC(16:0_22:0)	15.386336	18.263096	13.400904	21.837130	24.491248	22.322594	22.622211	20.488774	29.904488	--	--	--	1.15854685513549	1.4590963213746575	up
LIPID-N-0255	PC(16:0_20:3)	690.54465	600.99007	563.95471	889.59459	1094.5203	910.88879	974.77820	963.65864	1131.2184	--	--	--	1.17864902851166	1.5602372623676881	up
LIPID-N-0232	PC(16:0_18:2)	5698.0976	4813.7554	4740.5698	6175.2542	6642.9801	5712.8913	6326.8622	5970.1740	6305.7029	--	--	--	1.04291494135373	1.2149627504068023	up
LIPID-N-0276	PC(22:4_16:0)	156.80811	176.21262	143.50134	351.72100	399.87923	377.68559	326.45873	356.83799	427.84698	--	--	--	1.26441837130639	2.3698499174599794	up
LIPID-N-0984	PC(13:0_16:0)	44.097680	46.503795	33.948957	66.674846	89.620629	59.244799	51.957517	71.955792	73.025192	--	--	--	1.06718607480064	1.7305461710792371	up
LIPID-N-0981	PC(12:0_16:0)	60.514680	71.768717	45.228051	84.842623	75.857848	89.078787	83.132027	83.915747	90.833483	--	--	--	1.04115452838146	1.4071163276830412	up

PCs

Index	Compounds	p5-1	p5-2	p5-3	p21-1	p21-2	p21-3	TZ-1	TZ-2	TZ-3	TZ_vs_p21_VIP	TZ_vs_p21_Fold_Change	TZ_vs_p21_Type	p21_vs_p5_VIP	p21_vs_p5_Fold_Change	p21_vs_p5_Type
LIPID-N-0383	PE(16:0_20:4)	883.42599	956.91623	982.73297	1825.7273	1365.4412	1650.3908	1351.8150	1637.1413	1456.0237	--	--	--	1.19307503765835	1.714994856001102	up
LIPID-N-0389	PE(16:0_22:4)	273.12587	293.49321	255.73392	912.86366	1036.0014	882.32433	928.79809	1117.5695	842.25375	--	--	--	1.26813425535861	3.4427909518803457	up
LIPID-N-0397	PE(16:0_20:5)	31.140766	32.266748	21.329772	45.553686	46.273287	43.269862	40.002690	52.643406	40.432660	--	--	--	1.18129812778396	1.5943021192906204	up
LIPID-N-0401	PE(16:0_22:5)	131.04152	124.86589	119.49139	383.93971	394.46081	325.84639	371.51923	468.59494	338.24552	--	--	--	1.26453901790261	2.941530122132161	up
LIPID-N-0323	PE(16:0_16:0)	101.22589	106.77784	99.948410	144.08926	124.08176	117.96062	120.00807	118.61922	105.28171	--	--	--	1.09219972546373	1.2538690003746122	up
LIPID-N-0325	PE(16:0_18:0)	158.28049	166.29337	151.31854	316.81739	316.43559	278.23896	291.51385	319.58567	313.60512	--	--	--	1.26142937952979	1.915332013488574	up
LIPID-N-0334	PE(16:0_18:1)	2407.3358	2071.3735	2222.3166	3579.8575	3045.1507	2814.1279	2593.2778	3215.4632	2755.2449	--	--	--	1.14414634608109	1.4086106018218874	up
LIPID-N-1055	PE(16:0_17:1)	46.379864	46.620492	38.806784	60.231102	49.957811	50.675462	41.382093	43.722457	45.136737	2.0140132224014	0.8096341164568421	down	--	--	--
LIPID-N-0350	PE(18:2_16:0)	167.11475	167.46034	155.22713	272.06917	250.33090	259.19599	256.56897	286.25465	228.48373	--	--	--	1.27207684997998	1.5957380722765817	up
LIPID-N-0413	PE(22:6_16:0)	215.70318	228.72631	175.88686	636.31967	520.16810	516.27611	516.81636	578.39124	562.24918	--	--	--	1.25148430296593	2.6966302611852693	up
LIPID-N-0337	PE(20:1_16:0)	20.907748	23.047677	20.380541	46.717140	44.864498	37.556971	45.428342	43.918521	43.568711	--	--	--	1.23878486565606	2.0072537160768134	up
